# AAV8-Mediated Angiotensin-Converting Enzyme 2 Gene Delivery Prevents Experimental Autoimmune Uveitis by Regulating MAPK, NF-κB and STAT3 Pathways

**DOI:** 10.1038/srep31912

**Published:** 2016-08-25

**Authors:** Yiguo Qiu, Lifei Tao, Shijie Zheng, Ru Lin, Xinyu Fu, Zihe Chen, Chunyan Lei, Jiaming Wang, Hongwei Li, Qiuhong Li, Bo Lei

**Affiliations:** 1 Department of Ophthalmology, the First Affiliated Hospital of Chongqing Medical University, Chongqing Key Laboratory of Ophthalmology, Chongqing Eye Institute, Chongqing, China; 2 School of Biotechnology, Southern Medical University, Guangzhou, Guangdong China; 3 Department of Ophthalmology, University of Florida, Gainesville, Florida USA

**Keywords:** Eye diseases, Molecular medicine

## Abstract

Renin angiotensin system (RAS) is a key hormonal system which regulates the cardiovascular function and is implicated in several autoimmune diseases. With the discovery of the angiotensin-converting enzyme 2 (ACE2), a protective axis of RAS namely ACE2/Ang-(1–7)/Mas that counteracts the deleterious ACE/AngII/AT1R axis has been established. This axis is emerging as a novel target to attenuate ocular inflammation. However, the underlying molecular mechanisms remain unclear. We investigated the hypothesis that enhancing the activity of the protective axis of RAS by subretinal delivery of an AAV8 (Y733F)-ACE2 vector would protect against the ocular inflammation in experimental autoimmune uveitis (EAU) mice through regulating the local immune responses. Our studies demonstrated that increased ACE2 expression exerts protective effects on inflammation in EAU mouse by modulating ocular immune responses, including the differentiation of Th1/Th17 cells and the polarization of M1/M2 macrophages; whereas the systemic immune responses appeared not affected. These effects were mediated by activating the Ang-(1–7)/Mas and inhibiting the MAPK, NF-κB and STAT3 signaling pathways. This proof-of-concept study suggests that activation of ocular ACE2/Ang-(1–7)/Mas axis with AAV gene transfer modulates local immune responses and may be a promising, long-lasting therapeutic strategy for refractory and recurrent uveitis, as well as other inflammatory eye diseases.

**Electronic supplementary material:**

The online version of this article (doi:10.1038/srep31912) contains supplementary material, which is available to authorized users.

## Introduction

Uveitis is a sight-threatening intraocular inflammatory disease caused by autoimmune or infection-related immune responses. It is one of the main causes of severe visual impairment, even blindness within the working population worldwide^1^. Some uveitis is characterized by recurrence of inflammation that leads to progressive destruction of affected tissues, including the uveal tract, neural retina and adjacent tissues and consequently compromises the vision of patients. Thus, the refractory autoimmune disease is considered a tremendous challenge for treatment. The conventional treatment of uveitis includes corticosteroids, immunosuppressive agents and biologics^2^. However, serious potential side effects limit their use. Furthermore, a significant percentage of patients still do not respond well to these treatments^3^. Therefore, development of effective therapeutic approaches is important and urgent.

Experimental autoimmune uveoretinitis (EAU) is an animal model that shares many clinical and histological characteristics with human autoimmune uveitis^4^. It is a T cell mediated autoimmune disease model that closely resembles several types of uveitic disorders, such as Behçet’s disease as well as Vogt-Koyanagi-Harada disease^4,5^. EAU can be induced by immunizing the susceptible animals with a retinal antigen, such as interphotoreceptor retinoid-binding protein (IRBP), S-antigen, or by adoptive transfer of retinal antigen-specific CD4^+^ T cells^6,7,8^. Studies suggest that activated CD4^+^ T cells play an effector role in the ocular inflammatory pathological process in human uveitis and in EAU. It is now well recognized that two different T cell effector lineages: Th1 cells which predominantly produce the cytokine IFN-γ and Th17 cells in which the hallmark cytokine is IL-17, play crucial roles in uveitis^9^. More importantly, recurrence of autoimmune diseases may also be related to the activation of these T cell effector lineages. Experimental evidence shows that the relapsing/remitting of uveitis was attributed to the increased production of Th1 cytokine, suggesting that recurrent autoimmunity may originate to a degree of the autoreactive T cell^10^. In addition, treatment with mesenchymal stem cells downregulated Th1 and Th17 responses, then ultimately resulted in inhibiting the Th1/Th17-mediated inflammation and reduced the relapse of uveitis as well^11^.

The renin angiotensin system (RAS) was classically considered as a circulating hormone system which is essential in modulating the physiologic functions of the cardiovascular and renal systems. Dysfunction of RAS also attributes to the pathogenesis of inflammation and autoimmune diseases. Angiotensin II (Ang II) is the main peptide effector of RAS. It is a potent vasoconstrictor, growth modulator and pro-inflammatory molecule generated from the angiotensinogen through sequential enzymatic cleavages by renin and angiotensin converting enzyme (ACE)^12^. Ang II mediates deleterious effects primarily through a seven trans-membrane G-protein coupled receptor Ang II type 1 receptor (AT1R)^13^. Nevertheless, the linear cascade of the RAS has been dramatically modified recent years with many new components and pathways^14,15^. The most notable finding is the discovery of angiotensin-converting enzyme 2 (ACE2). ACE2 cleaves Ang II to generate Angiotensin-(1–7) [Ang-(1–7)] which mediates counteracting physiologic functions to Ang II via a G-protein coupled receptor Mas. Consequently, a concept of a novel antagonist axis of RAS, i.e. the ACE2/Ang-(1–7)/Mas axis is established^16^. Increasing evidence have demonstrated that activation of this axis not only directly counterbalances the deleterious effects of the classical ACE/Ang II/AT1R axis, but also has a broad range of beneficial effects including ameliorating pathological conditions such as fibrosis and inflammation^17,18^.

The ACE2/Ang-(1–7)/Mas axis has protective effects in many tissues and organs^18^. ACE2/Ang-(1–7)/Mas axis acts as a negative regulator of the RAS and has become a vital therapeutic target in many inflammatory and autoimmune diseases, including type 1 diabetes^19^, autoimmune myocarditis^20^ and arthritis^21^. Moreover, it is protective in several ocular immune diseases. Increased expression of ACE2 and Ang-(1–7) via AAV-mediated gene delivery to the retina diminished retinopathy and inflammation in both mouse and rat models of diabetic retinopathy^22^. Activation of ACE2 is neuroprotective in a glaucoma animal model^23^. More recently, we demonstrated that activating the endogenous ACE2 both systemically and locally evoked beneficial effects by attenuating the inflammation of endotoxin induced uveitis (EIU) in mice and rats^24,25^. Furthermore, oral delivery of ACE2/Ang-(1–7) bioencapsulated in plant cells conferred protection against ocular inflammation in EAU and EIU mice^26^. Thus, enhancing the ACE2/ Ang-(1–7)/Mas axis of the RAS may be a promising therapeutic approach for ocular inflammation.

However, the underlying mechanisms by which over-expression of ACE2 confer protection against ocular autoimmune inflammation remain poorly understood. We previously showed that the hyper-activation of the nuclear factor (NF)-κB and mitogen activated protein kinase (MAPK) signaling pathways were involved in the pathological process of EAU and EIU^25,27^. Expression of signal transducer and activator of transcription 3 (STAT3) in CD4^+^ T cells is essential for the development of EAU and mice with conditional deletion of STAT3 are completely resistant to EAU^28^. On the other hand, ACE2 activator DIZE mediates anti-inflammatory properties by down-regulating phosphorylation of p38MAPK, extracellular signal-regulated kinase (ERK) and c-Jun N-terminal kinase (JNK), STAT3 and NF-κB p65 subunit in macrophages^29^. Over-expression of ACE2 significantly inhibits the development of early atherosclerotic lesions in a rabbit model by down-regulating the p38MAPK, ERK1/2 and STAT3 pathways^30^. Hence, we hypothesize that over-expression of ACE2 attenuates the ocular local inflammation in EAU mouse by activating the protective ACE2/Ang-(1–7)/Mas axis and by inhibition of MAPK, NF-κB and STAT3 pathways. We employed adeno-associated virus vector serotype 8 (AAV8) vector containing a tyrosine-capsid mutant (Y733F) to deliver the *ACE2* gene subretinally since this capsid-modified vector has been shown to provide rapid and efficient gene transduction in adult mouse retina^31^.

## Results

### Subretinal administration of AAV8 (Y733F) vectors successfully transducted the ACE2 and enhanced green fluorescent protein (eGFP) in the retinas

**Figure 1:**
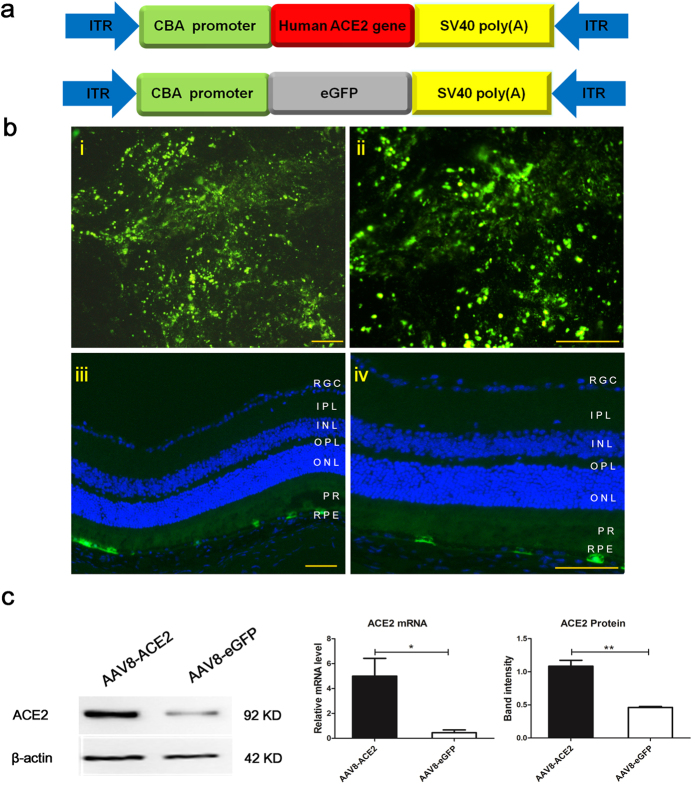
Construction and characterization of AAV8 (Y733F) vector expressing ACE2 and enhanced green fluorescent protein (eGFP). **(a)** Maps of the AAV8(Y733F) vector expressing the human *ACE2* gene *(hACE2*) and a control vector contains coding region for the secreted enhanced green fluorescent protein (eGFP). CBA, CMV-chicken-β-actin promoter; ITR, inverted terminal repeat. (**b**) Transduction of mouse retina with AAV8 (Y733F) vector expressing eGFP. A single subretinal injection of 1 μl AAV8 (Y733F) vector (10^11^ vg/ml) resulted in robust expression of eGFP. (i) A retinal whole mount showing the expression of eGFP in the magnification of 100X. (ii) Higher magnification of the same retinal whole mount (200X). (iii) A cross section of a mouse eye showed eGFP expression at three weeks after receiving AAV8(Y733F)-eGFP injection in low magnification of 100X. The eGFP was mainly transducted at the RPE and the PR level of the retina. (iv) Higher magnification of the same cross section (200X). Scale bar = 100 μm. RPE, retinal pigment epithelium; PR, photoreceptor; ONL, outer nuclear layer; OPL, outer plexiform layer; INL, inner nuclear layer; IPL, inner plexiform layer; RGC, retinal ganglion cells. (**c**) The mRNA and protein expression of ACE2 in the eyes injected with AAV8(Y733F)-ACE2 or AAV8(Y733F)-eGFP vectors. Data were shown as mean ± SEM (*p < 0.05, **p < 0.01, n = 4).

AAV8 vectors containing a secreted form of human *ACE2* gene or enhanced green fluorescent protein (eGFP) under the control of the chicken β-actin (CBA) promoter were constructed as shown in Fig. 1a. A single subretinal injection of 1 μl AAV8 (Y733F) vector (10^11^ vg/ml) resulted in robust expression of eGFP. The flatmount image showed 3 weeks after the injection, the fluorescence was strongly expressed, especially around the site where the vector was injected, which was in consistent with previous studies^31^. Previous studies demonstrated that AAV8-Y733F vector mediated gene delivery would result in gene expression mainly in the RPE layer and photoreceptor layer^31,32^, which was in parallel with our findings as shown in Fig. 1b. The cross section showed that the eGFP was mainly transducted at the RPE and the photoreceptor level of the retina. Three weeks after subretinal injection of AAV8-ACE2 vector, the expression of the target gene ACE2 was increased at both mRNA level and protein level (Fig. 1c) (*p < 0.05).

### Subretinal administration of AAV8-ACE2 decreased the clinical and histological scores of EAU

**Figure 2:**
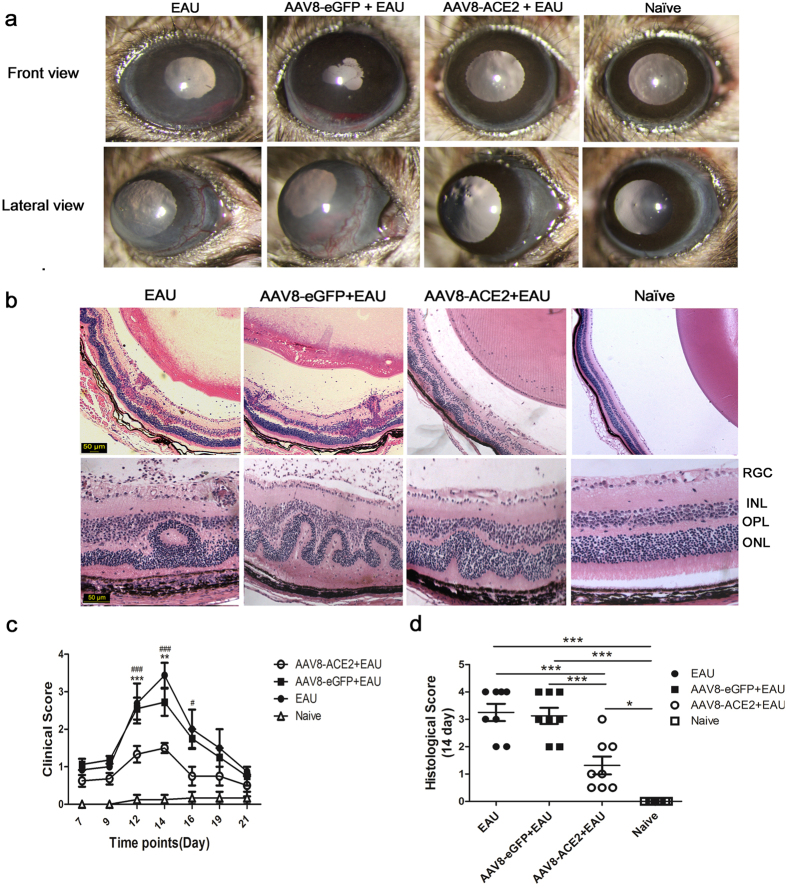
Clinical and histological evaluation of EAU mice. Subretinal delivery of AAV8-ACE2 reduced the clinical and histological scores in EAU mice. **(a)** Clinical signs were assessed with a slit lamp from day 7 to day 21 after IRBP immunization. Representative images showed the anterior inflammation from the naïve mice, AAV8-ACE2 or AAV8-eGFP treated EAU mice and EAU mice at the 14^th^ day after immunization. Corneal edema, conjunctival hyperemia, hypopyon and posterior synechiae were seen in the AAV8-eGFP+EAU and EAU group. Whereas there were mild inflammatory signs in the AAV8-ACE2+EAU group. **(c)** The severity of clinical scores were remarkably decreased on the 12^th^, 14^th^ and 16^th^ day in the AAV8-ACE2 treated eyes compared with the AAV8-eGFP treated EAU eyes (**P < 0.01, ***P < 0.001) and EAU eyes (^#^*P* < 0.05, ^###^*P* < 0.001, n = 4–8 per group). **(b)** Histological scores were assessed by hematoxylin and eosin (H&E) staining paraffin-embedded sections of eyes collected at the 14^th^ day after immunization. Representative images from the AAV8-eGFP treated EAU mice and EAU mice showed severe retinal folds, damage of the photoreceptor layer and massive infiltrated inflammatory cell in the vitreous, retina and subretinal space; minor infiltration of cells and retinal folding was observed in the AAV8-ACE2+EAU group. **(d)** The AAV8-ACE2 treated EAU group showed a reduced EAU histological scores compared to the AAV8-eGFP treated EAU group (***p < 0.001) and EAU group (***p < 0.001). The independent experiment was repeated for 4 times. Magnification: 50×, 200×, scale bar = 50 μm. Data were shown as mean ± SEM (n = 8 per group).

The anterior chamber of EAU mice and the control vector (AAV8-eGFP) treated mice showed severe inflammatory responses, including corneal edema, conjunctival hyperemia, hypopyon and posterior synechiae at the 14^th^ day after IRBP immunization. The severity of inflammation was remarkably attenuated in the AAV8-ACE2 treated eyes when compared with the AAV8-eGPF treated eyes of the EAU mice (Fig. 2a). The clinical score was significantly decreased in the AAV8-ACE2 treated eyes of the EAU mice at the 12^th^ and 14^th^ day when compared with AAV8-eGPF treated EAU eyes and the eyes of EAU mice (Fig. 2c). To evaluate the histological scores, eyes were collected at the 14^th^ day after IRBP immunization. Over-expression of ACE2 significantly reduced the histological damages (Fig. 2b). Histological examination showed a severe intraocular inflammation as evidenced by massive infiltration of inflammatory cells, folds of retina and photoreceptor damage in the AAV8-eGFP + EAU mice and EAU mice. Whereas, only very few scattered inflammatory cells and minor retinal folds were observed in the AAV8-ACE2 treated EAU mice (Fig. 2b). The histological scores evaluated according to the criteria reported previously^33^, were significantly decreased in the AAV8-ACE2 treated EAU mice (1.125 ± 0.629) as compared to the AAV8-eGFP treated controls (3 ± 0.816) and EAU mice (3.25 ± 0.957) (Fig. 2d).

### Administration with AAV8-ACE2 rescued retinal function loss in EAU mice

**Figure 3:**
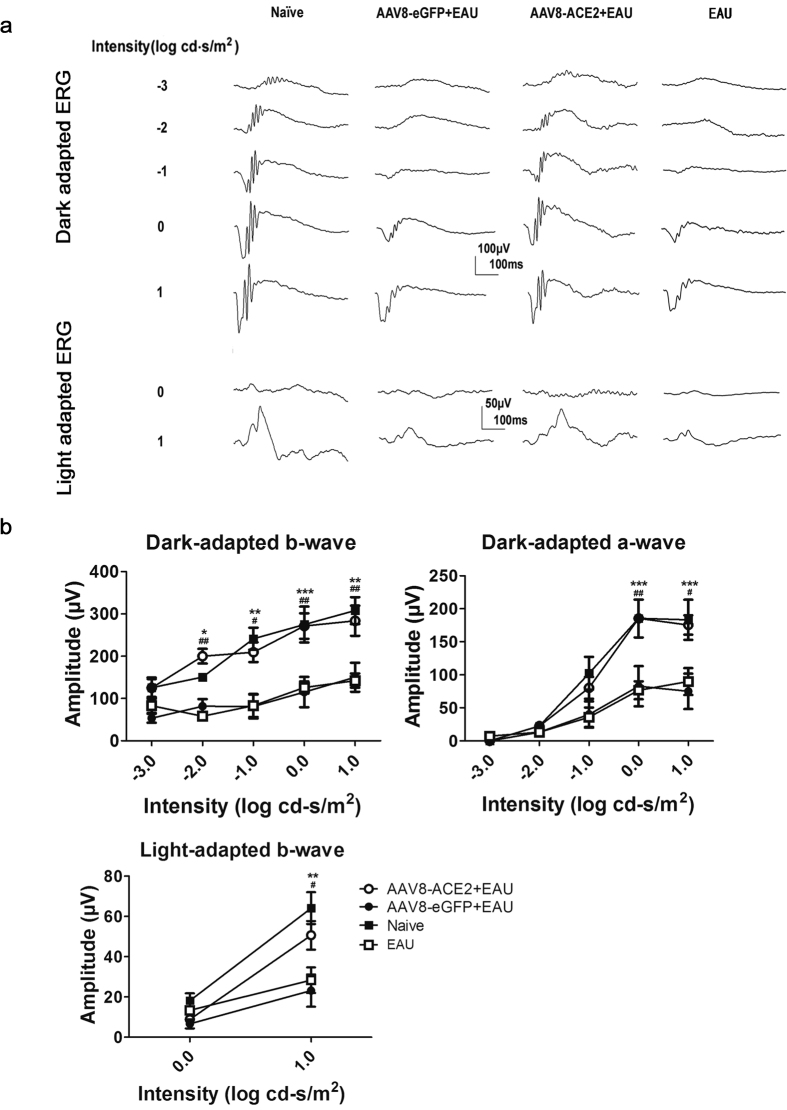
Effects of AAV8-mediated ACE2 delivery on retinal function in EAU mice. To determine the effects of over-expression of ACE2 on retinal function, both dark-and light-adapted ERG were recorded in EAU mice at the 14^th^ day after immunization. For dark-adapted ERG, the stimulus light intensity ranged from −3.0 to 1.0 (log cd-s/m2). For light-adapted ERG, the stimulus light intensities were 0.0 and 1.0 (log cd-s/m2). **(a)** Representative ERG responses in the AAV8-ACE2+EAU, AAV8-eGFP+EAU, EAU and naïve groups. **(b)** Dark and light-adapted ERG amplitudes vs. intensity profile was shown. Data were shown as mean ± SEM (*p < 0.05, **p < 0.01, ***p < 0.001 versus AAV8-eGFP+EAU group; #p < 0.05, ##p < 0.01, versus EAU group; n = 7~10 per group).

There was a remarkable damage of retinal photoreceptor layer and destruction of the normal structure of retina at the 14^th^ day after immunization. Therefore, to detect the effect of AAV8-mediated ACE2 over-expression on retinal dysfunction in EAU, both dark- and light-adapted ERG was performed at the 14^th^ day after IRBP immunization. The amplitudes of a- and b-wave represent the function of the photoreceptors and bipolar cells respectively. Representative original ERG responses from AAV8-ACE2 + EAU, AAV8-eGFP+EAU, EAU and naïve group were shown (Fig. 3a). ERG responses of naïve mice were used as controls. The amplitudes of the dark-adapted ERG a- and b-wave as well as light-adapted ERG b-wave were significantly preserved in the AAV8-ACE2 treated EAU group compared with the AAV8-eGFP +EAU group (*p < 0.05, **p < 0.01, ***p < 0.001) and untreated EAU group (#*p* < 0.05, ##*p* < 0.01) (Fig. 3b).

### Subretinal delivery of AAV8-ACE2 reduced the expression of the inflammatory cytokines in EAU mice

**Figure 4:**
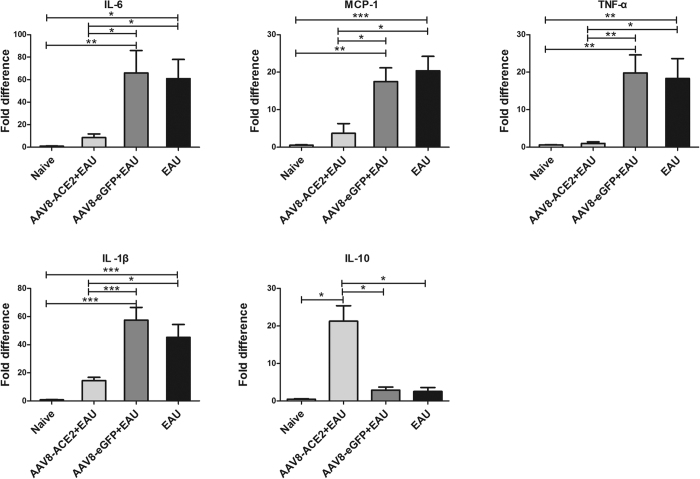
Real-time PCR analysis of retinal mRNA expression for inflammatory cytokines in the AAV8-ACE2 treated EAU mice. Retinas were collected for analyzing the mRNA levels of the pro-inflammatory cytokines IL-6, IL-1β, TNF-α, MCP-1 and anti-inflammatory cytokine IL-10. Values on y-axis represent fold difference of the mRNA expressions at the 14^th^ day after IRBP immunization. The mRNA levels of the pro-inflammatory cytokines were significantly decreased, whereas the expression of IL-10 was remarkably increased in the AAV8-ACE2+EAU group compared with the AAV8-eGFP+EAU and the EAU groups (*p < 0.05, **p < 0.01, ***p < 0.001). Data were expressed as mean ± SEM (n = 4–6 per group).

To investigate the effect of ACE2 on the expression of pro-inflammatory and anti-inflammatory cytokines in EAU eyes, total RNA was isolated from the retina on the 14^th^ day after IRBP immunization. Real-time PCR was performed to quantitatively measure the levels of the inflammatory cytokines. Compared with the AAV8-eGFP+EAU and EAU group, the mRNA levels of interleukin-6 (IL-6), interleukin-1β (IL-1β), tumor necrosis factor-α (TNF-α) and monocyte chemoattractant protein-1 (MCP-1) were markedly decreased, whereas the expression of IL-10 was significantly increased in the AAV2-ACE2 treated eyes. (Fig. 4). In the AAV8-ACE2 + EAU group, there was approximately a 7.8-fold decrease in the mRNA level of IL-6 (*p* < 0.05) and approximately a 4-fold decrease of IL-1β (*p*<0.001). The mRNA levels of TNF-α and MCP-1 were also significantly reduced in the AAV8-ACE2 treated EAU eyes. There was approximately a 19-fold decrease of TNF-α (*p* < 0.01) and a 4.7-fold decrease of MCP-1 (*p* < 0.05) compared with the AAV8-eGFP+EAU group. The mRNA levels of IL-10 was about 7.4-fold increased compared to AAV8-eGFP+EAU group (Fig. 4).

### AAV8-ACE2 inhibited the Th1/Th17 responses and shifted the polarization of M1 to M2 macrophages locally, but not systemically

**Figure 5:**
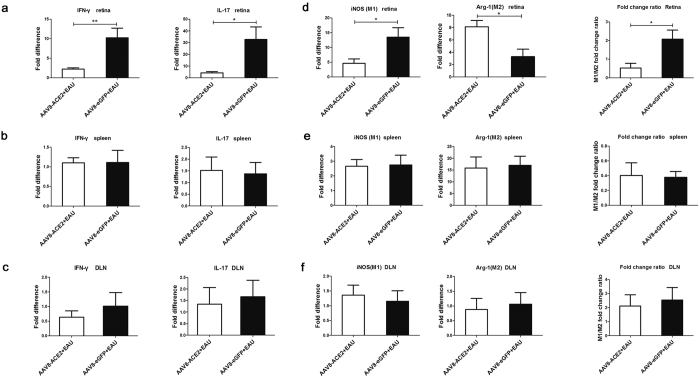
The effect of ACE2 over-expression on the local and systemic mRNA expression of IFN-γ, IL-17 and polarization of M1/M2 macrophages. The retinas, spleens and draining lymph nodes (DLN) were harvested for analyzing the mRNA of IFN-γ and IL-17, the representative cytokines for Th1 and Th17 cells respectively, iNOS (the marker of M1 macrophages) and Arginase-1 (Arg-1, the marker of M2 macrophages). Values on y-axis represent fold difference of the mRNA expressions at the 14^th^ day after IRBP immunization. The mRNA levels of IFN-γ and IL-17 in the retinas (**a**), spleens (**b**) and draining lymph nodes (**c**) (*p < 0.05, **p < 0.01, n = 4~6 per group). (**d**) The retinal mRNA level of iNOS was decreased and the level of Arg-1 was increased in the AAV8-ACE2+EAU group compared with the AAV8-eGFP+EAU group. The M1/M2 fold change ratio was significantly increased in the AAV8-eGFP+EAU group (*p < 0.05, n = 4~6 per group). However, the expression of iNOS or Arg-1 was no statistical difference between the two groups in spleens (**e**) and draining lymph nodes (**f**). Data were expressed as mean ± SEM.

Th1 and Th17 cells play essential roles in the process of EAU^9^. The expressions of IFN-γ and IL-17 indicate the activity of Th1/Th17 responses. To determine the effect of ACE2 over-expression on the local and systemic Th1/Th17 responses, retinas, spleens and draining lymph nodes (DLN) were collected to detect the mRNA levels of IFN-γ and IL-17. A significant decrease of IFN-γ and IL-17 at the mRNA level in the retinas was observed in the AAV8-ACE2+EAU group compared with the AAV8-eGFP + EAU group (*p* < 0.05, *p* < 0.01) (Fig. 5a). Nevertheless, there was no significant difference of IFN-γ and IL-17 between the AAV8-ACE2 delivered EAU group and the AAV8-eGFP + EAU group in spleens (Fig. 5b) or DLN (Fig. 5c).

Macrophages are considered to be important effectors in EAU^34^ via the production of inflammatory and immunomodulatory cytokines^35^. Shifting of the macrophage subtypes may affect the the process of EAU. Decrease of M1 subtype but increase of M2 subtype may confer beneficial effects to EAU^36,37^. Thus, to investigate whether ACE2 would regulate the polarization of the macrophage subtype locally and systemically, typical surface markers of M1 (iNOS) and M2 (Arginase-1) macrophages were detected by real-time PCR. In the retina, the mRNA level of iNOS was increased in the AAV8-eGFP+EAU group, whereas, the level of Arginase-1 (Arg-1) was decreased compared with the ACE2 over-expression EAU eyes (*p* < 0.05). The M1/M2 ratio was higher (2.08 ± 0.84) in the AAV8-eGFP+EAU mice than in the AAV8-ACE2 + EAU mice (0.53 ± 0.50) (Fig. 5d). Nevertheless, there was no statistical difference in iNOS or Arg-1 level between the AAV8-ACE2 + EAU and the AAV8-eGFP + EAU groups in the spleen (Fig. 5e) and DLN (Fig. 5f). Thus ACE2 administration may shift the polarization of M1 to M2 macrophages locally in the retina, but not systemically.

### A-779, an Ang-(1–7) antagonist, abolished the anti-inflammatory effect of ACE2 in EAU and abrogated the regulatory effect on the differentiation of Th1/Th17 cells and polarization of M1/M2 macrophages

**Figure 6:**
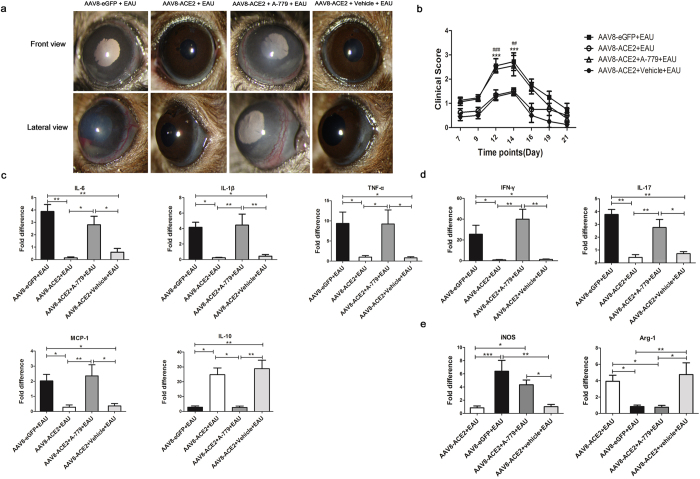
The effect of A-779 on the clinical scores, retinal mRNA expression of the cytokines and the surface markers of M1/M2 macrophages. (**a**) Clinical signs were assessed with a slit lamp from day 7 to day 21 after the mice received IRBP immunization and the implantation of mini-pumps with A-779 or vehicle. Representative images showed the anterior inflammation from the AAV8-ACE2 or AAV8-eGFP treated EAU mice and AAV8-ACE2 treated EAU mice with the A-779 or vehicle implantation at the 14^th^ day after immunization. (**b**) The severity of clinical scores were remarkably decreased on the 12^th^, 14^th^ day in the AAV8-ACE2 treated eyes compared with the AAV8-eGFP treated EAU eyes (***P < 0.001) and AAV8-ACE2+A-779+EAU eyes (^##^*P* < 0.01, ^###^*P* < 0.001, n = 4–8 per group). The retinas were dissected at the 14^th^ day after IRBP immunization for analyzing the mRNA levels of the pro-inflammatory and anti-inflammatory cytokines and the surface markers of M1/M2 macrophages. (**c**) The mRNA levels of pro-inflammatory cytokines: IL-6, IL-1β, TNF-α and MCP-1 and anti-inflammatory cytokine: IL-10 in the AAV8-ACE2+EAU, AAV8-eGFP+EAU, AAV8-ACE2+EAU+A-779 and AAV8-ACE2+vehicle+EAU groups. (**d**) The mRNA expression of Th1 cell signature cytokine IFN-γ and Th17 cell signature cytokine IL-17 were remarkably reduced in the AAV8-ACE2 treated EAU eyes compared with the AAV8-eGFP treated eyes. A-779 abrogated the reduction of IFN-γ and IL-17. (**e**) The mRNA of iNOS was decreased, whereas the mRNA of Arg-1 was increased in the AAV8-ACE2+EAU group. However, A-779 reversed the effect of ACE2 on the polarization of M1/M2 macrophages. Data were expressed as mean ± SEM (*p < 0.05, **p < 0.01, ***p < 0.001, n = 4–6 per group).

ACE2 cleaves Ang II to generate Ang-(1–7), which activates its downstream signaling pathways via the G-protein coupled receptor Mas. To confirm that the protective effects of ACE2 over-expression in EAU eyes is mediated by Ang-(1–7)/Mas signaling, we used the Ang-(1–7) antagonist A-779. Co-administration of A-779 via mini-pump reversed the protective effects of ACE2 on the inflammatory signs and the production of inflammatory cytokines as well as the regulation of the local immune responses. As shown in Fig. 6a, the severity of inflammatory signs in AAV8-ACE2 treated EAU eyes were milder than AAV8-eGFP+EAU group. However, A-779 abrogated this protective effect of ACE2, as evidenced by more severe inflammation and higher clinical scores of AAV8-ACE2+A-779+EAU group (Fig. 6a,b). The mRNA expressions of IL-6, IL-1β, TNF-α and MCP-1 were remarkably decreased and the level of IL-10 was increased in the AAV8-ACE2+EAU group. However, with the co-administration of A-779, the reduction of these pro-inflammatory cytokines and the increase of the anti-inflammatory cytokine was reversed (*p* < 0.05, *p* < 0.01), indicating decreased pro-inflammatory cytokine production and increased anti-inflammatory cytokine by ACE2 over-expression was mediated through Ang-(1–7)/Mas (Fig. 6c). Subretinal administration with AAV8-ACE2 also inhibited the Th1/Th17 responses as evidenced by a significant decrease of IFN-γ and IL-17. However, A-779 abrogated the reduction of IFN-γ and IL-17 (*p* < 0.05, *p* < 0.01) (Fig. 6d). Moreover, A-779 abolished the effects of ACE2 over-expression on shifting the polarization of M1/M2 macrophages, as demonstrated by negating the expression of iNOS and Arg-1. (Fig. 6e). Therefore, the protective effects of ACE2 over-expression were mediated by activating Ang-(1–7)/Mas.

### The inhibitors of MAPK, NF-κB and STAT3 alleviated the Th1 and Th17 responses in the IRBP-induced inflammation in lymphocytes

**Figure 7:**
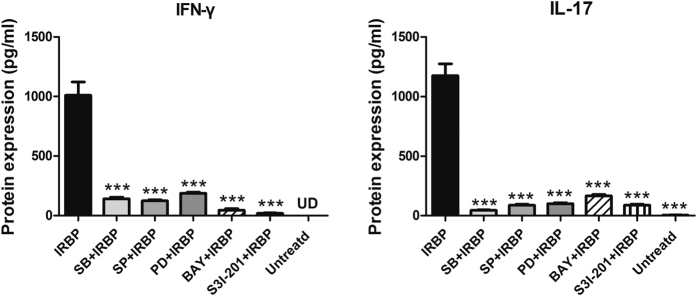
The expression of IFN-γ and IL-17 in response to IRBP stimulation with or without MAPK, NF-κB or STAT3 inhibitors. Lymphocytes were extracted from the spleens and draining lymph nodes of EAU mice. The lymphocytes were pre-treated with or without the MAPK, NF-κB or STAT3 inhibitors for 30 minutes and then incubated with IRBP for 72 hours. The protein expression levels of IFN-γ and IL-17 were measured by ELISA 72 hours after stimulation by IRBP with or without the presence of the p38 MAPK inhibitor (SB203580, SB), JNK inhibitor (SP600125, SP), ERK1/2 inhibitor (PD98059, PD), NF-κB inhibitor (BAY11-7082, BAY) at a final concentration of 10 μM and STAT3 inhibitor S3I-201 at the concentration of 100 μM. The data were shown as mean ± SEM (***p < 0.001, n = 8~10). UD: undetected.

To elucidate whether the MAPK, NF-κB and STAT3 pathways were involved in IRBP-induced inflammation, lymphocytes of spleens and draining lymph nodes were collected from EAU mice and pre-treated with MAPK, NF-κB or STAT3 pathway inhibitors, followed by IRBP incubation for 72 hours. Inhibition of p38 MAPK with the highly selective inhibitor (SB20358046, SB) reduced IRBP-induced production of IFN-γ and IL-17 at the protein level by approximately 7.58-, 22.67-fold, respectively. A JNK inhibitor (SP600125, SP) decreased protein expression of IFN-γ and IL-17 by 8.76- and 12.83-fold, respectively. Inhibition of ERK 1/2 with the inhibitor (PD98059, PD) reduced IFN-γ and IL-17 expression by approximately 1.88-, 11.41-fold. The NF-κB inhibitor (BAY11-7082, BAY) resulted in 29.47- and 6.39-fold reductions of IFN-γ and IL-17 respectively at the protein level. Moreover, the inhibitor of STAT3, S3I-201 decreased the protein level of IFN-γ and IL-17 by 70.29-, 11.34-fold, respectively (*p* < 0.001, versus IRBP group) (Fig. 7). Fold changes of protein levels are shown in Table S1.

### The anti-inflammatory effect of activating ACE2/Ang-(1–7)/Mas axis in the EAU eyes is associated with the inhibition of MAPK, NF-κB and STAT3 pathways

**Figure 8:**
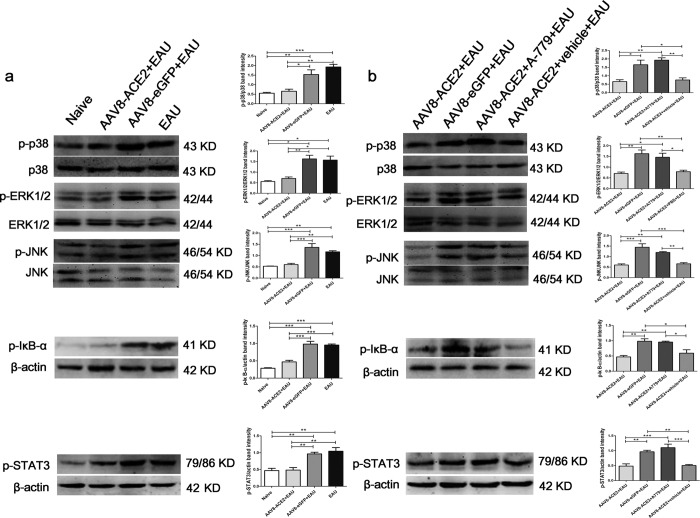
Involvement of the MAPK, NF-κB and STAT3 pathways in the ACE2/Ang-(1–7)/Mas axis mediated protection in EAU eye. **(a)** The phosphorylation levels of p38 MAPK, ERK1/2, JNK, IκB-α and STAT3 (p-p38 MAPK, p-ERK1/2, p-JNK, p-IκB-α and p-STAT3) in the AAV8-ACE2+EAU group compared with the AAV8-eGFP+EAU and EAU groups were determined by Western blotting (*p < 0.05, **p < 0.01, ***p < 0.001, n = 4~6). The band intensities of p-p38 MAPK, p-ERK1/2 and p-JNK were normalized to p38 MAPK, ERK1/2 and JNK, respectively. The band intensities of p-IκB-α and p-STAT3 were normalized to β-actin. **(b)** The protein expression of p-p38 MAPK, p-ERK1/2, p-JNK, p-IκB-α and p-STAT3 in the AAV8-ACE2+EAU group compared with the AAV8-ACE2+EAU+A-779 and AAV8-ACE2 +EAU+vehicle groups was determined by Western blotting. Relative expression of p-p38 MAPK, p-ERK1/2 and p-JNK were normalized to p38 MAPK, ERK1/2 and JNK, respectively. The relative expression of p-IκB-α and p-STAT3 were normalized to β-actin. The data were shown as mean ± SEM (*p < 0.05, **p < 0.01, ***p < 0.001, n = 4~6).

To further determine whether inhibition of the MAPK, NF-κB and STAT3 pathways is involved in the ACE2/Ang-(1–7)/Mas axis mediated protection in EAU eyes, phosphorylation level of p38 MAPK, ERK1/2, JNK, IκB-α and STAT3 in the retina were measured by Western blotting at the 14^th^ day after immunization. The phosphorylation levels of all the five proteins were increased in the EAU group (Fig. 8), however, were significantly decreased in the AAV8-ACE2 treated EAU group compared with the AAV8-eGFP treated EAU and EAU groups (*p* < 0.05, *p* < 0.01, *p* < 0.001) (Fig. 8a). We further confirmed that the protective effect of inhibiting these pathways is mediated by activating Ang-(1–7)/Mas. The Ang-(1–7) antagonist, A-779, abolished the protective effect. The phosphorylation levels of p38 MAPK, ERK1/2, JNK, IκB-α and STAT3 were remarkably increased in the AAV8-ACE2 + EAU mice received A-779 mini-pump implantation compared to the AAV8-ACE2 + EAU mice (*p* < 0.05, *p* < 0.01, *p* < 0.001). However, there were no significant difference of the phosphorylation levels between the AAV8-ACE2+EAU group and the AAV8-ACE2 + EAU received vehicle mini-pump implantation group (Fig. 8b).

## Discussion

In addition to the circulating system, most components of the RAS have been identified in numerous organs including the eye^38^. Indeed, ACE2 was detected in porcine ciliary bodies and retinas^39^ and Ang-(1–7) and Mas were also observed in the eyes^40,41,42,43^. These studies provide solid evidence that the ACE2/Ang-(1–7)/Mas axis of the RAS exists in ocular tissues. The tissue-specific RAS is believed to exert diverse physiological effects locally independent of circulating RAS^44^. Thus, in-depth studies to elucidate the physiopathologic functions of the local RAS in different organs are highly desirable.

Here we demonstrated that subretinal delivery of the *ACE2* gene resulted in a robust expression of ACE2 at both mRNA and protein levels in the retina. Over-expression of ACE2 significantly reduced the effector lineage of T cells, as well as shifted the phenotype of macrophages in the retina. However, subretinal delivery of ACE2 is incapable to affect the responses in spleens and DLNs, indicating that the activation of the local ACE2/Ang-(1–7)/Mas axis may be capable to modulate the local immune responses, thus to intervene the pathological process of EAU.

It has been shown recently that activation of the protective the ACE2/Ang-(1–7)/Mas axis exhibits anti-inflammatory actions in several animal models of ocular diseases including diabetic retinopathy^22^ and endotoxin-induced uveitis^24^. In agreement with these studies, we found over-expression of ACE2 via AAV8 (Y733F) gene delivery exerted protective effects in the EAU mouse model, as evidenced by ameliorating the clinical signs, suppressing the tissue damage and improving the retinal neuronal function. The anti-inflammatory effect of ACE2 appeared to result in reducing the expression of inflammatory cytokines and influence the ocular immune responses. The antagonist of Ang-(1–7) A-779 abolished these beneficial effects, suggesting that these protective effects are mediated by activating the ACE2/Ang-(1–7)/Mas axis. Interestingly, increased ACE2 expression might be capable to modulate the ocular local immune responses, including reducing the expression of IFN-γ and IL-17 and shifting the phenotype of macrophages from M1 to M2. We suggest inhibition of MAPK, NF-κB and STAT3 signaling pathways are involved in these protective effects of enhancing ACE2.

T cell effector lineages, especially Th1 and Th17 cells contribute to the immune pathogenesis of EAU^45,46^. Th1 and Th17 responses have also been reported in uveitis patients^47^. Recent studies illustrated a major role for the RAS in autoimmunity. Treatment with the ACE inhibitor lisinopril or with the AT1R antagonist candesartan resulted in the suppression of Th1 and Th17 cytokines (specifically IFN-γ and IL-17) in a murine model of human multiple sclerosis^48^. It has been shown that blockade of the classical RAS axis alleviated the brain inflammation by reducing the expression of transforming growth factor (TGF)-β in a model of autoimmune encephalomyelitis (EAE)^49^ and attenuates the clinical symptoms of rheumatoid arthritis by the modulation of the T cell cytokines profile^50^. In addition, Activation of the Mas receptor by Ang-(1–7) significantly ameliorated arthritis in murine models^21^. These results suggest that the RAS system is critically involved in promoting Th1/Th17-mediated autoimmune diseases in different organs.

In line with the previous studies, we showed that activating the ACE2/Ang-(1–7)/Mas axis by over-expressing ACE2 resulted in a dramatic decrease of the typical Th1 and Th17 cytokines (IFN-γ and IL-17), indicating that enhancing ACE2 may ameliorate EAU by inhibiting local Th1 and Th17 responses. Although there was a evidence that NK and NKT cells may be attribute to in part the production of IFN-γ in EAU, it was just during the innate response phase, thus to shape the effector T cells^51^. In addition, by using immunohistochemical staining and *in situ* hybridization, a previous study demonstrated that the presence of IFN-γ was only localized to the T lymphocytes infiltrated in the retina and uveal tract in the EAU model. Notably, when the inflammatory cells infiltrated in the posterior chamber, no IFN-γ has been detected^52^. Similarly, despite NKT cells can produce IL-17, it is also happens in the innate response which differs significantly from adaptive IL-17 produced by Th17 effector cells. Moreover, it is produced only by a relatively small NK1.1-negative fraction. As a consequence, its amount is very low and can hardly be detected^53^. Therefore, we strongly believe the expression of IFN-γ and IL-17 in the retina can serve as indicators of Th1 and Th17 response in EAU model. Nevertheless, further study are necessary for investigating the possible role of these two signature cytokines in different immune cells and different stages of EAU. It may shed light on potential novel therapeutic targets to protect against uveitis in further studies.

In addition to the T cells, it has been shown that activation of ACE2/Ang-(1–7)/Mas axis reduces inflammatory responses in macrophages^54^. Macrophages are considered to be potent antigen presenting cells (APCs) in inflammatory autoimmune disease and are responsible for the production of inflammatory and immunomodulatory cytokines^35^. They also play an essential role in tissue damage during the process of autoimmune diseases, including arthritis, nephritis and experimental allergic encephalomyelitis (EAE)^55,56^. It has been reported that macrophages are important at different stages of EAU^34^. In particular, they are the major effectors of tissue damage during EAU^34^. Macrophages affect the T cell responses depending on different levels of cytokines and chemokines in the microenvironment^57^. The heterogeneity of macrophage has been well established^58^. They are engaged in versatile activities in parallel with the Th1 and Th2 types of immune responses, which have been termed type 1 (M1) and type 2 (M2) macrophages^58^. M1 macrophages are classically activated macrophages, which develop in response to inflammatory stimuli of Th1 cytokine, such as IFN-γ. Activated M1 macrophages produce large amounts of proinflammatory cytokines such as TNF-α, IL-1β and IL-6. Whereas M2 macrophages are alternatively activated macrophages and are considered to be anti-inflammatory macrophages^59^. A shift of macrophages from the M1 to M2 phenotype reduces the experimental inflammatory colitis^60^. Downregulating the M1/M2 ratio of macrophage has been shown to attenuate the Behçet’s disease (BD)-like symptoms in a herpes simplex virus (HSV)-induced inflammatory mouse model^36^. Further, IL-33 increases the frequency of M2 macrophages in the lymphoid organs confers a protective effect in EAU^37^.

In consistent with the previous studies, our results showed that the mRNA level of the proinflammatory cytokines produced by the M1 macrophages, including TNF-α, IL-1β and IL-6 dramatically decreased in the AAV8-ACE2 treated EAU eyes compared with the AAV8-eGFP treated EAU eyes. Meanwhile, the mRNA expression of the M1 marker iNOS was significantly decreased. On the contrary, the M2 markers increased in the AAV8-ACE2 treated EAU eyes compared with the AAV8-eGFP treated EAU eyes. Furthermore, we found a down-regulated M1/M2 ratio with the treatment of AAV8-ACE2 in the EAU retinas. However, subretinal injection with AAV8-ACE2 or AAV8-eGFP did not influence the polarization of macrophages in spleens or DLNs. These result revealed that activating the local protective ACE2/Ang-(1–7)/Mas axis may be capable to regulate the polarization of classically activated M1 macrophage and the alternatively activated M2 macrophage in the eyes of EAU mice. Shifting of the phenotype of macrophages may have an impact on ocular inflammation in EAU. However, more in-depth researches to verify this possibility need to be done in the future works.

Importantly, in the process of inflammation, a cascade of intracellular signaling pathways will be initiated and ultimately lead to activation of macrophages and release of pro-inflammatory cytokines. The major intracellular events include the phosphorylation of MAPK, STATs pathways and NF-κB, the crucial transcription factor for proinflammatory cytokine production^61,62^. It has been suggested that these signaling pathways were involved in the ACE2/Ang-(1–7)/Mas axis mediated protection. Indeed, by modulating Ang-(1–7) and Mas receptor, an ACE inhibitor olmesartan exerts cardioprotective and anti-inflammatory effects in the experimental autoimmune myocarditis model through decreasing the phosphorylation of p38MAPK, extracellular signalregulated kinase (ERK) and jun N-terminal kinase (JNK) pathways^63^. In addition, in an allergic asthma mouse model, Ang-(1–7) ameliorated inflammation which was attributed to one of the MAPK family members, the ERK and NF-κB signaling pathway^64^. Most recently, it was shown DIZE, an activator of endogenous ACE2, remarkably suppressed LPS induced production of proinflammatory cytokines. DIZE downregulated the phosphorylation of key signaling molecules and transcription factors including MAPKs, STATs and NF-κB p65 subunit, which are involved in the production of proinflammatory cytokines^29^.

Consistent with previous studies, we found over-expression of ACE2 alleviated the ocular inflammation not only by reducing the inflammatory cytokines, but also by decreasing the phosphorylation of MAPK, NF-κB and STAT3 pathways. Although activating the ACE2/Ang-(1–7)/Mas axis has been documented in animal models of diabetic retinopathy^22^ and glaucoma^23^, to our knowledge, no research has been performed in the classical ocular inflammatory diseases and autoimmune ocular inflammation. Besides, the underlying mechanisms of the advantageous effects mediated by enhancing the ACE2/Ang-(1–7)/Mas axis in ocular inflammation remains poorly understood. We provide the first piece of evidence that the activation of ACE2 plays an anti-inflammatory role in ocular autoimmune inflammation by regulating the local immune responses and the protective effects are associated with the inhibition of MAPK, NF-κB and STAT3 pathways.

Many uveitis patients are prone to relapse, the prolonged inflammation often results in a chronic refractory inflammation. After the conventional medications, patients still suffer from recurrent inflammation, suggesting that the treatments can not completely eliminate the pathogenic factors. We found that activating the ACE2/Ang-(1–7)/Mas axis effectively suppressed the Th1/Th17 responses which not only contribute to the primary inflammation process of uveitis, but also may play an important role in the recurrent inflammation in autoimmune uveitis^11^. Therefore, it is practical to provide a long-lasting strategy to enhance the protective axis of the RAS in these uveitis patients. Moreover, long-term application of the conventional treatments, such as corticosteroids and immunosuppressants, may cause various systemic and local unwanted side effects. Thus topical administration has an advantage of less risk of potential off-target side effects. AAV vector mediated gene therapy for ocular diseases has been successfully applied in human and animal for more than a decade^65^. In view of recent clinical trials^66^, the safety of AAV mediated gene therapy of ocular diseases has been warranted. Furthermore, AAV-associated gene therapies were shown to be highly effective. Existing evidences showed that AAV-mediated ACE2 gene delivery ameliorated the inflammatory responses in diabetic retinopathy^22,67^. Moreover, oral delivery of ACE2 or Ang-(1–7) vector resulted in down-regulation of inflammatory cytokines in EIU and EAU models^26^. Meanwhile a single AAV vector injection yields a long-term target gene expression which is ideal for treating the chronic and recurrent uveitis. Therefore, AAV mediated ACE2 delivery may be a promising novel intervention for uveitis, as well as other chronic ocular inflammatory conditions.

However, there are some limitations in our study. First, although the safety of systemic ACE2 activator application^24^ and AAV mediated gene therapy of ocular diseases has been warranted in several clinical trials^66^ and we did not observed any systemic and ocular side effects in our study, it is unknown whether long-term ocular ACE2 over-expression would cause any unwanted systemic or local adverse effects. Hence, a long-term safety study of local ACE2 over-expression should be carried out. Second, to ensure the transferred gene to be functional, we delivered the vectors three weeks before inducing the EAU. Thus enhanced ACE2 occurred before the on-set of inflammation and our study described a prophylactic effect of enhancing ACE2 in the inflammatory process. Nevertheless, in most cases, since it takes a few weeks for the transferred gene to be functional and since it is impossible to treat the patients preventively, we can’t modify the ACE2/Ang-(1–7)/Mas axis for the initial uveitis patients by gene therapy in a timely manner. However, a combination of an ACE2 activator, such as DIZE together with gene transfer may be a practical option for these initial patients. Theoretically, it appears that this issue is not a problem for the recurrent patients.

In summary, we demonstrated that activation of the ACE2/Ang-(1–7)/Mas axis by subretinal delivery of AAV8(Y733F)-ACE2 conferred protection against the ocular inflammation in EAU mice. Of significant interest, the protective axis is limited to modulate the local immune responses, including the differentiation of Th1/Th17 cells and the polarization of M1/M2 macrophages. In contrast to systemic administration, local gene delivery of the ACE2 may significantly reduce the off-target risks. In addition, gene delivery achieves a long-term activation of the RAS protective axis and thus may provide a novel therapeutic strategy for refractory and recurrent uveitis as well as other ocular autoimmune inflammatory diseases.

## Materials and Methods

### Ethics statement

This study was carried out according to the ARVO Statement for the Use of Animals in Ophthalmic and Vision Research. The protocols were approved by the Ethics Committee of the First Affiliated Hospital of Chongqing Medical University. All surgeries were performed under anesthesia and all efforts were made to minimize animal discomfort and stress.

### Animals and experimental procedures

B10.RIII mice were purchased from the Jackson Laboratory (Bar Harbor, ME). All animals were housed under specific pathogen-free conditions at the Animal Care Service of Chongqing Medical University with a 12–12-hour light-dark cycle. Mice with the age of 6 to 8 weeks were randomly divided into four groups: (1) AAV8-ACE2 [short for AAV8(Y733F)-CBA-ACE2] injected EAU group: mice were induced EAU at three weeks after subretinal injection of AAV8-ACE2 vector; (2) AAV8-eGFP [short for AAV8(Y733F)-CBA-eGFP] injected EAU group: mice were induced EAU at three weeks after subretinal injection of AAV8-eGFP vector; (3) EAU group and (4) naïve group.

To study whether the effect of AAV8-ACE2 was mediated by activating of Ang-(1–7)/Mas, osmotic mini-pumps (Alzet, Cupertino, CA, USA) filled with a selective Ang-(1–7) antagonist, A-779 (D-Ala7-ANG I/II/1–7) with the delivery rate of 125 μg·kg^−1^·h^−1^ (Bachem, Torrence, CA, USA) were implanted subcutaneously on the back between the shoulder blades and hips while animals were anesthetized by intraperitoneal injection with 75 mg/kg ketamine (Fujian Gutian pharmaceutical Co., Ltd, Ningde, Fujian, China) and 13.6 mg/kg xylazine (VEDCO Inc., St. Joseph, MO, USA). Control mice were sham operated the same way as mice that were implanted with osmotic minipumps containing vehicle (sterile water). The mini-pumps filled with A-779 or vehicle were implanted at the same time when IRBP_161–180_ was given.

### Subretinal injections

One microliter of vector (~10^8^ vector genome) of AAV8-eGFP or AAV8-ACE2 was injected subretinally into one eye. Subretinal injection was performed according to the method described previously^33^. All procedures were performed under antiseptic conditions. Briefly, the eyes were dilated by topical administration of compound tropicamide eye drops (Sinqi Pharmaceutical Co., Ltd., Shenyang, China). An aperture within the dilated pupil area was made through the cornea with a 30-gauge needle, then a blunt 33-gauge needle was inserted through the aperture, avoiding damage the lens and penetrating the neuroretina under a dissecting microscope (Leica, DMR, Deerfield, IL,USA). The successful delivery of vector was confirmed by viewing fluorescein-positive subretinal blebs demarcating the retinal detachment in the injected retinal area. Such detachments usually can be resolved spontaneously within 1 to 2 days. The damages occasionally induced by ocular injection included temporal corneal edema, iris hemorrhage or cataract formation. The animals with any of these complications were excluded from further study. All animals received antibiotic ointment to the cornea and were observed daily after operation. The vectors were injected subretinally into the right eyes of the mice, the lateral eyes were not injected and served as a control.

### Induction of EAU

Human interphotoreceptor retinoid binding protein (IRBP) peptide 161-180 (IRBP161–180, SGIPYIISYLHPGNTILHVD) was synthesized by Shanghai Sangon Biological Engineering Technology & Services Ltd. Co. (Shanghai, China). Complete Freund’s adjuvant (CFA) was obtained from Sigma-Aldrich (St. Louis, MO, USA). EAU was induced as a previous protocol^33^. Briefly, mice were immunized subcutaneously at the base of the tail and both thighs with 50 μg human IRBP 161–180 peptide in 100 μl PBS, emulsified 1:1 (vol/vol) in CFA (Sigma-Aldrich, St. Louis, MO, USA) supplemented with 1.0 mg/ml mycobacterium tuberculosis strain. A total 200 μl emulsion was given in one mouse.

### Clinical and histological assessment of EAU

Clinical signs of EAU were examined by slit-lamp microscopy from day 7 to day 21 after immunization. The clinical severity of ocular inflammation was assessed by two independent observers in a masked manner and scored on a scale of 0–5 in half-point increments, according to five separate criteria described previously^33^. To evaluate the histopathology changes, mice were sacrificed at the 14^th^ day after IRBP peptide immunization. Eyes were enucleated and fixed in 4% paraformaldehyde (PFA) at 4 °C overnight. Eyeballs were embedded in paraffin. Serial 4 μm sections were cut through the cornea-optic nerve axis and stained by haematoxylin and eosin (H&E). For histopathologic evaluation, the severity of EAU was graded in a masked fashion on a scale of 0 to 4, as described earlier^33^.

### Electroretinogram (ERG) analysis

Both dark- and light-adapted ERG (RetiMINER System; AiErXi Medical Equipment Co., Ltd, Chongqing, China) were recorded at the 14^th^ day after IRBP immunization. The procedures were performed as previous described^7^. Briefly, mice were dark-adapted overnight and anesthetized with the mixture of ketamine and xylazine. The pupils were dilated with 0.5% tropicamide and 0.5% phenylephrine HCl. Active electrodes were gently placed on the center of cornea. Reference and ground electrodes were inserted to the back neck and near the tail subcutaneously. All procedures were performed under dim red light. The amplitude of the a-wave was measured from the baseline to the peak of a-wave and the b-wave was measured from the nadir of the a-wave to the apex of the b-wave peak.

### Real time PCR analysis

Total RNA was extracted from the freshly enucleated retinas, spleens and draining lymph nodes (LN) by using Trizol Reagent (Invitrogen, Carlsbad, CA, USA) according to the manufacturer’s instructions. Complementary DNA (cDNA) was generated using PrimeScript RT reagent Kit (Takara Biotechnology, Dalian, China). Real-time PCR was performed according to manufacturer’s instruction with the real-time PCR system (ABI Prism 7500; Applied Biosystems, Foster City, CA, USA). Each reaction was run in duplicate, Relative quantification was achieved by the comparative 2^−ΔΔCt^ method as described previously^24^. Real time-PCR was performed in a volume of 20 μl by using SYBR Premix Ex TaqTM II (Takara Biotechnology, Dalian, China). The conditions were 95 °C for 10 min, followed by 40 cycles of 15 s at 95 °C and 60 s at 60 °C. Amplification of specific transcripts was confirmed by melting curve profiles at the end of each PCR. Primer sequences used in this study are shown in Table S2.

### Immunofluorescence staining

For immunofluorescence staining, the eyeballs were enucleated and fixed in 4% paraformaldehyde (PFA) and phosphate buffer saline (PBS, 0.01 M phosphate buffer, 0.0027 M potassium chloride and 0.137 M sodium chloride, pH 7.4) then cryoprotected in 30% sucrose overnight. Then eyes were embedded in optimal cutting temperature (OCT) compound (Sakura Finetek Inc., Torrance, CA, USA). Serial 10 μm sections were cut through the cornea-optic nerve axis and stained with 2-(4-Amidinophenyl)-6-indolecarbamidine dihydrochloride (DAPI, Beyotime, Shanghai, China) for 5 minutes at room temperature. After three times’ wash, the frozen sections were viewed under a digital camera using a Leica fluorescence microscope (DMR, Deerfield, IL, USA).

### The signal transduction mechanism analysis with inhibitors of p38 MAPK, ERK1/2, JNK, NF-κB and STAT3 after IRBP immunization by enzyme-linked immunosorbent assay (ELISA)

For investigating the signal transduction mechanism involving MAPK, NF-κB and STAT3 pathways with the corresponding inhibitors on IRBP immunized lymphocytes, the spleens and draining lymph nodes were removed from EAU mice on the 14^th^ day after immunization. Cell suspension was prepared by mechanical disruption and followed by a passage through a cell strainer (BD, Franklin Lakes, NJ, USA). The lymphocytes were separated by using a specific lymphocyte separation kit (TBDscience, Tianjin, China). The RBC lysis buffer was applied to the cells after gradient centrifugation with the Ficoll-hypaque separation buffer, so that the contamination of RBC could be excluded. Although the lymphocytes are a subset of leukocytes, we can still detect the expression of IFN-γ and IL-17 which are mainly produced by Th1 and Th17 cells, rather than other leukocytes^9,68^, to reflect the severity of EAU. The lymphocytes were seeded into a 24-well plate (2~3*10^5^ per well) and pre-incubated with p38MAPK inhibitor SB203580 (Cell Signaling Technology, Danvers, MA, USA), ERK1/2 inhibitor PD98059 (Cell Signaling Technology, Danvers, MA, USA), JNK inhibitor SP600125 (Cell Signaling Technology, Danvers, MA, USA), NF-κB inhibitor BAY11-7082 (Sigma-Aldrich, St. Louis, MO, USA) at the concentration of 10 μM and STAT3 inhibitor S3I-201 (Santa Cruz Biotechnology, Inc., Santa Cruz, CA, USA) at the concentration of 100 μM for 30 minutes. Then the cells were cultured with RPMI 1640 medium (Gibco, Grand Island, NY, USA) and 10% fetal bovine serum in the presence of 10 μg/mL IRBP161–180 peptide for 72 hours. After 72 hours’ incubation, the supernatants were collected for detecting the concentrations of IFN-γ and IL-17 by the mouse ELISA Duoset kits (R&D Systems, Minneapolis, MN, USA) according to the manufacturer’s instructions.

### Western Blotting Analysis

The retinas were collected 14 days after IRBP immunization and lysed by Radio Immuno Precipitation Assay (RIPA) Lysis Buffer (Beyotime, Shanghai, China) including 1% proteases inhibitor (Beyotime, Shanghai, China). The lysate was centrifuged and the supernatant was collected. Protein concentration was determined by bicinchoninic acid (BCA) protein kit (Beyotime, Shanghai, China). All samples were diluted in SDS loading buffer (Beyotime, Shanghai, China) and boiled for 5 minutes. Equal amounts of protein (80 μg) were loaded to 10% polyacrylamide gel for the SDS-PAGE electrophoresis. The protein was then transferred to nitrocellulose membranes (Millipore, Billerica, MA, USA). Membranes were blocked with 5% skim milk or 5% bovine serum albumin (BSA) and incubated with specific primary antibodies against mouse ACE2 (1:200, Santa Cruz Biotechnology, Inc., Santa Cruz, CA, USA), p-IκBα (1:50, Santa Cruz Biotechnology, Inc., Santa Cruz, CA, USA), β-actin(1:100, ABCAM, Cambridge, MA, USA), p38, JNK, ERK1/2, phosphorylated p38 (p-p38), p-ERK1/2 (1:1000, Cell Signaling Technology, Danvers, MA, USA), JNK, p-JNK (1:500, Cell Signaling Technology, Danvers, MA, USA), STAT3, p-STAT3 (Tyr705) (1:1000, Cell Signaling Technology, Danvers, MA, USA) over night at 4 °C, followed by the secondary antibody (1:3000, ABCAM, Cambridge, MA, USA) at 37 °C for 1 hour. The membranes were further developed by Western Bright^TM^ ECL kit (Advansta, Menlo Park, CA, USA). Bands were analyzed using Image J software (Version1.43, Broken Symmetry Software, Bethesda, MD, USA). Analysis was normalized against a housekeeping protein β-actin. The band intensity of p-p38, p-ERK1/2 and p-JNK was normalized to p38, ERK1/2 and JNK respectively.

### Statistical Analysis

All data were expressed as mean ± SEM. Statistical analysis was performed with the GraphPad Prism 5 software (GraphPad Software, Inc., San Diego, CA, USA). Two-tailed tests were used in this study. Experimental data were analyzed by one-way ANOVA followed by Bonferroni correction for multiple group comparisons. Unpaired Student’s t-test was used to assess significance between two groups. Clinical scores were analyzed by two-way ANOVA followed by Bonferroni correction. A Mann-Whitney *U* test was used to compare the histological scores of EAU. *p* < 0.05 was considered statistically significant.

## Additional Information

**How to cite this article**: Qiu, Y. *et al.* AAV8-Mediated Angiotensin-Converting Enzyme 2 Gene Delivery Prevents Experimental Autoimmune Uveitis by Regulating MAPK, NF-κB and STAT3 Pathways. *Sci. Rep.* **6**, 31912; doi: 10.1038/srep31912 (2016).

## Electronic supplementary material

Supplementary Information
