# Supplementary material for: AAV8-Mediated Angiotensin-Converting Enzyme 2 Gene Delivery Prevents Experimental Autoimmune Uveitis by Regulating MAPK, NF-κB and STAT3 Pathways
Source: Sci Rep. 2016 Aug 25;6:31912. doi: 10.1038/srep31912 (PMC4997264; doi:10.1038/srep31912)
Supplement: Supplementary file 1 — Supplementary Information [file 41598_2016_BFsrep31912_MOESM1_ESM.pdf]

AAV8-Mediated Angiotensin-Converting Enzyme 2 Gene Delivery Prevents Experimental Autoimmune Uveitis by Regulating MAPK, NF-κB and STAT3 Pathways

Yiguo Qiu[1], Lifei Tao[1], Shijie Zheng[1], Ru Lin[1], Xinyu Fu[1], Zihe Chen[1], Chunyan Lei[1], Jiaming Wang[1], Hongwei Li[2], Qiuhong Li[3], Bo Lei[1]

[1] Department of Ophthalmology, the First Affiliated Hospital of Chongqing Medical University, Chongqing Key Laboratory of Ophthalmology, Chongqing Eye Institute, Chongqing, China
[2] School of Biotechnology, Southern Medical University, Guangzhou, Guangdong, China
[3] Department of Ophthalmology, University of Florida, Gainesville, Florida, USA

Supplementary Methods:

Recombinant adeno-associated virus construction and packaging

AAV vectors containing a secreted form of human *ACE2* gene or enhanced green fluorescent protein (eGFP) under the control of the chicken β-actin (CBA) promoter were constructed as previous studies[1, 2]. The secreted ACE2 protein has been proved to be enzymatically activated in the preceding study[3]. These constructs contained expression cassettes flanked by the rAAV8 terminal repeats. Expressions of eGFP and ACE2 were driven by a CBA with a human cytomegalovirus enhancer. Site-directed mutagenesis of surface-exposed tyrosine residues on the capsids to generate AAV8(Y733F) mutation has been described recently[4]. Vector plasmid was packaged in AAV8 (Y733F) by transfection of HEK cells according to previously published

methods[5]. Vector doses were expressed as genome copies.

References

1. Verma, A. et al. ACE2 and Ang-(1-7) confer protection against development of diabetic retinopathy. *Mol Ther* **20**,28-36 (2012).
2. Li, H. et al. Macrophage migration inhibitory factor in hypothalamic paraventricular nucleus neurons decreases blood pressure in spontaneously hypertensive rats. *Faseb J* **22**,3175-3185 (2008).
3. Huentelman, M.J., Zubcevic J., Katovich M.J. & Raizada M.K. Cloning and characterization of a secreted form of angiotensin-converting enzyme 2. *Regul Pept* **122**,61-67 (2004).
4. Petrs-Silva, H. et al. High-efficiency transduction of the mouse retina by tyrosine-mutant AAV serotype vectors. *Mol Ther* **17**,463-471 (2009).
5. Zolotukhin, S. et al. Recombinant adeno-associated virus purification using novel methods improves infectious titer and yield. *Gene Ther* **6**,973-985 (1999).

Supplementary Table S1

Table S1. Fold changes of IFN-γ and IL-17 at the protein level (VS. IRBP group)

group	cytokines	protein fold change
SB+IRBP	IFN-γ	7.58
	IL-17	22.67
SP+IRBP	IFN-γ	8.76
	IL-17	12.83
PD+IRBP	IFN-γ	1.88
	IL-17	11.41
BAY+IRBP	IFN-γ	29.47
	IL-17	6.39
S3I-201+IRBP	IFN-γ	70.29
	IL-17	11.34

Supplementary Table S2

Table S2. Primers used for Real-Time PCR analysis

Gene name	Accession number	Sequences
ACE2	NM_001130513.1	Forward: 5'-GATGTCATTCCTAGAAGTGAAG-3'
		Reverse: 5'-ACATACAGGAAGATGACAAGTG-3'
IL-6	NM_031168.1	Forward: 5'-AGATAACAAGAAAGACAAAGCCAGAGTC-3'
		Reverse: 5'-GCATTGGAAATTGGGGTAGGAAG-3'
IL-1β	NM_008361.3	Forward: 5'- TTGAAGAAGAGCCCGTCC-3'
		Reverse: 5'-CTTATGTTCTGTCCATTGAGGT-3'
TNF-α	NM_001278601.1	Forward: 5'-AGGCGCCACATCTCCCTCCA-3'
		Reverse: 5'-CGGTGTGGGTGAGGAGCACG-3'
MCP-1	NM_011333.3	Forward: 5'-AGTTGCCGGCTGGAGCATCC-3'
		Reverse: 5'-TCTTTGGGACACCTGCTGCTGG-3 '
IL-10	NM_010548.2	Forward: 5'-GAAGACCCTCAGGATGCG-3'
		Reverse: 5'-CCAAGGAGTTGTTTCCGTTA-3 '
IFN-γ	NM_008337.3	Forward: 5'-TCAAGTGGCATAGATGTGGAAGAA-3'
		Reverse: 5'-TGGCTCTGCAGGATTTTCATG-3'
IL-17	NM_010552.3	Forward: 5'-CTCAACCGTTCCACGTCACCCT-3'
		Reverse: 5'-CCAGCTTTCCCTCCGCATT-3'
iNOS	NM_010927.3	Forward: 5'-GGGCTGTCACGGAGATCA-3'
		Reverse: 5'-CCATGATGGTCACATTCTGC-3'
Arg-1	NM_007482.3	Forward: 5'-GAACACGGCAGTGGCTTTAAC-3'
		Reverse: 5'-TGCTTAGCTCTGTCTGCTTTGC-3'
GAPDH	NM_001289726.1	Forward: 5'-GTATGACTCCACTCACGGCAAA-3'
		Reverse: 5'-GGTCTCGCTCCTGGAAGATG-3'
